# A dengue receptor as possible genetic marker of vector competence in *Aedes aegypti*

**DOI:** 10.1186/1471-2180-8-118

**Published:** 2008-07-15

**Authors:** Ricardo F Mercado-Curiel, William C Black, Maria de L Muñoz

**Affiliations:** 1 Department of Genetics and Molecular Biology, Centro de Investigación y de Estudios Avanzados del Instituto Politécnico Nacional. Ave. Instituto Politécnico Nacional 2508 Col San Pedro Zacatenco, C.P. 07360, México, DF, México; 2 Department of Microbiology, Colorado State University, Fort Collins 80523, USA

## Abstract

**Background:**

Vector competence refers to the intrinsic permissiveness of an arthropod vector for infection, replication and transmission of a virus. Notwithstanding studies of Quantitative Trait Loci (QTL) that influence the ability of *Aedes aegypti *midgut (MG) to become infected with dengue virus (DENV), no study to date has been undertaken to identify genetic markers of vector competence. Furthermore, it is known that mosquito populations differ greatly in their susceptibility to flaviviruses. Differences in vector competence may, at least in part, be due to the presence of specific midgut epithelial receptors and their identification would be a significant step forward in understanding the interaction of the virus with the mosquito. The first interaction of DENV with the insect is through proteins in the apical membrane of the midgut epithelium resulting in binding and receptor-mediated endocytosis of the virus, and this determines cell permissiveness to infection. The susceptibility of mosquitoes to infection may therefore depend on their specific virus receptors. To study this interaction in *Ae. aegypti *strains that differ in their vector competence for DENV, we investigated the *DS3 *strain (susceptible to DENV), the *IBO-11 *strain (refractory to infection) and the membrane escape barrier strain, *DMEB*, which is infected exclusively in the midgut epithelial cells.

**Results:**

(1) We determined the MG proteins that bind DENV by an overlay protein binding assay (VOPBA) in *Ae. aegypti *mosquitoes of the *DS3*, *DMEB *and *IBO-11 *strains. The main protein identified had an apparent molecular weight of 67 kDa, although the protein identified in the *IBO-11 *strain showed a lower mass (64 kDa). (2) The midgut proteins recognized by DENV were also determined by VOPBA after two-dimensional gel electrophoresis. (3) To determine whether the same proteins were identified in all three strains, we obtained polyclonal antibodies against R67 and R64 and tested them against the three strains by immunoblotting; both antibodies recognized the 67 and 64 kDa proteins, corroborating the VOPBA results. (4) Specific antibodies against both proteins were used for immunofluorescent location by confocal microscopy; the antibodies recognized the basal lamina all along the MG, and cell membranes and intercellular spaces from the middle to the end of the posterior midgut (pPMG) in the neighborhood of the hindgut. (5) Quantitative analysis showed more intense fluorescence in *DS3 *and *DMEB *than in *IBO-11*. (6) The viral envelope antigen was not homogeneously distributed during MG infection but correlated with MG density and the distribution of R67/R64.

**Conclusion:**

In this paper we provide evidence that the 67 kDa protein (R67/R64), described previously as a DENV receptor, is related to vector competence in *Ae. aegypti*. Consequently, our results strongly suggest that this protein may be a marker of vector competence for DENV in *Ae. aegypti *mosquitoes.

## Background

DENV is a Flavivirus within the Arboviruses class, more than 500 of which have so far been identified. DENV is distributed worldwide in tropical and subtropical countries in association with its mosquito vector *Ae. aegypti*. Dengue infection ranges from self-limited asymptomatic or mild illness (dengue fever, DF) to a severe hemorrhagic disease (dengue hemorrhagic fever, DHF) that can progress to dengue shock syndrome (DSS) characterized by circulatory failure [1].

More than fifty million dengue infection cases occur every year [2], resulting in approximately 24,000 deaths due to DSS. In Mexico [3], 45,748 cases of DF and 10,501 of DHF were reported from 2004 to 2006. Although DEN is the most common vector-borne viral disease, few studies have investigated the complex relationship between DENV and *Ae. aegypti *through their genetic characteristics.

Transmission of infection depends on DENV virulence, host immunity and the susceptibility of the mosquito to infection. Susceptibility will depend on the interaction between the mosquito and DENV: the interaction between midgut (MG) cell membrane receptors and the virus envelope glycoprotein is the initial step [4] in receptor-mediated endocytosis [5,6]. This essential step determines cell permissiveness to infection. Furthermore, it is known that populations of *Ae. aegypti *differ greatly in their susceptibility to DENV [7-10] and this variability is determined by the effects of several genes [11,12]. It has also been proposed [7] that the infection barriers in the mosquitoes are the MG infection barrier (MIB), which prevents DENV infection of MG epithelial cells, and the MG escape barrier (MEB), which prevents DENV from leaving the MG and infecting peripheral tissues, limiting the infection to MG cells. Three *Ae. aegypti *strains differing in DENV vector competence were selected: the susceptible *DS3 *strain lacks MIB and MEB, so 95–100% of these mosquitoes have disseminated infection [13]; the *DMEB *strain has MEB, and although 85% of them are infected in the MG epithelial cells, 27% develop a disseminated infection [13]; and the refractory strain, *IBO-11*, has MIB [14]. Differences in vector competence may, at least in part, be due to the presence of specific MG epithelial receptors and their identification would be a significant step forward in understanding the interaction of the virus with the mosquito vector with possible implications for vector surveillance and control of virus transmission.

Fc gamma receptor-mediated entry of infectious DENV immune complexes into human monocytes/macrophages is hypothesized to be a key event in the pathogenesis of DHF [15-18]. However, this mechanism does not explain virus entry in primary infections or in cells with non-Fc receptors such as those of mosquitoes [19,20].

Putative non-Fc gamma receptors differ in chemical structure [21]; proteins [22-34] or glycoproteins [35]; heparan sulfates [36] and LPS/CD14-associated binding proteins [37] have been proposed as cellular receptors for DENV. We have previously shown that the four serotypes of DENV mainly recognize two proteins with apparent molecular weights of 80 and 67 kDa [24,29] from *Ae. albopictus *C6/36 cells and *Ae. aegypt*i MG. Although a variety of receptors have been described in many studies, the mechanism by which the virus enters the cell is unknown.

A study of receptors in the *DS3*, *DMEB *and *IBO-11 *strains will allow us to determine whether they are related to the susceptibility of *Ae. aegypti *to DENV infection and transmission, and consequently whether they may serve as genetic markers of vector competence. To date, none have been described. Knowledge of one or more genes responsible for susceptibility will help in designing new control strategies that may prevent DENV infection and dissemination in the mosquito vector [38].

Therefore, the objective of this work was to determine whether DENV receptors from *Ae. aegypti *MG are related to vector competence. The aim was to identify the proteins recognized by DENV in three *Ae. aegypti *strains that differ in their susceptibility to infection.

## Results

### Identification of DENV binding proteins in MG from *DS3*, *DMEB *and *IBO-11 *by VOPBA

**Figure 1:**
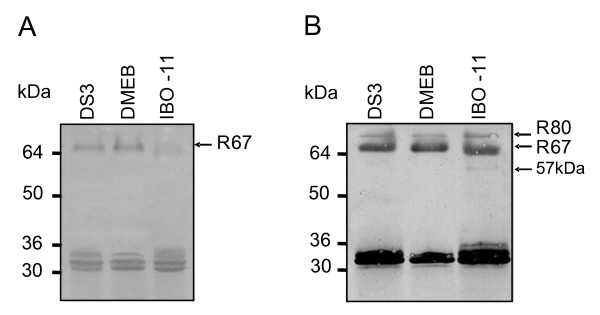
**VOPBA of membranes blotted from SDS-PAGE of MG protein extract from *DS3*, *DMEB *and *IBO*-11 strains**. A. 35 μg of protein were loaded on each lane; actin was used as control for the amount of protein loaded. B. The amounts of protein loaded on each lane were: 72 μg for *DS3*; 59 μg for *DMEB*; 155 μg for *IBO-11*.

To determine the apparent molecular weights of DENV-binding MG proteins from each *Ae. aegypti *strain, equivalent amounts of MG protein extracts (35 μg) were separated by SDS-PAGE, blotted on to PVDF membranes and analyzed by VOPBA (Figure 1A) using the same specific antibodies against dengue virus used in our previous study [29]. In the *IBO-11 *strain, DENV bound mainly to a protein of apparent molecular weight about 64 kDa, whereas in the *DS3 *and *DMEB *strains the apparent molecular mass of this protein was 67 kDa. Proteins with molecular masses of 80 and 57 kDa were also visualized, as previously described [29], though these bands were not very strong. Because the *IBO-11 *strain showed a very faint band, we repeated the experiment, increasing the protein concentration approximately 3-fold compared to *DMEB *(Figure 1B). The masses of protein loaded on to the gel for each strain were: 72 μg for *DS3*, 59 μg for *DMEB *and 155 μg for *IBO-11*. DENV recognized the protein with apparent molecular mass of 80 kDa with approximately the same intensity in all three strains; the 67 kDa protein had the same molecular weight in the *DS3 *and *DMEB *strains, but in the *IBO-11 *strain the apparent molecular mass was 64 kDa. As a control for gel loading accuracy, blots were probed with anti-actin antibody; each specific band on the membranes was quantified by densitometry and normalized to actin. The amount of DENV bound to the 67/64 kDa proteins in each strain varied in the proportions *DMEB*: *DS3*: *IBO-11 *= 77: 56: 1. These differences may be due to differences in the amount of MG protein from each mosquito strain or in their affinity for DENV.

**Figure 2:**
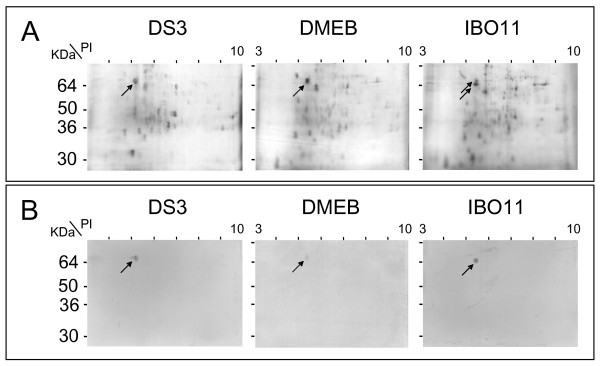
**Two dimensional gel electrophoresis analysis of proteins from *DS3*, *DMEB *and *IBO-11 *strains**. A. Two dimensional gel electrophoresis of *DS3*, *DMEB *and *IBO-11 *protein extracts (300 μg). B. VOPBA of PVDF membranes blotted from two dimensional gel electrophograms of MG protein extracts from these strains. Black arrows indicate the dots that were recognized by DENV.

To determine whether the proteins identified by VOPBA represent one or more different molecules or whether there are differences among the strains, 300 MGs were subjected to two-dimensional gel electrophoresis (Figure 2A) and a VOPBA was performed on the gel (Figure 2B). In the *DS3 *and *DMEB *strains, DENV mainly recognized two protein spots with almost the same apparent molecular mass of 67 kDa (black arrow). The *IBO-11 *strain displayed the same spots, although the lower one, which was more evident, probably corresponds to the protein with apparent molecular mass about 64 kDa. The faint upper spot may correspond to the protein with molecular mass of 67 kDa. The isoelectric point of both proteins in the three strains was about 5.3.

### Identification of R67 and R64 in *DS3*, *DMEB *and *IBO-11 *by immunoblotting

**Figure 3:**
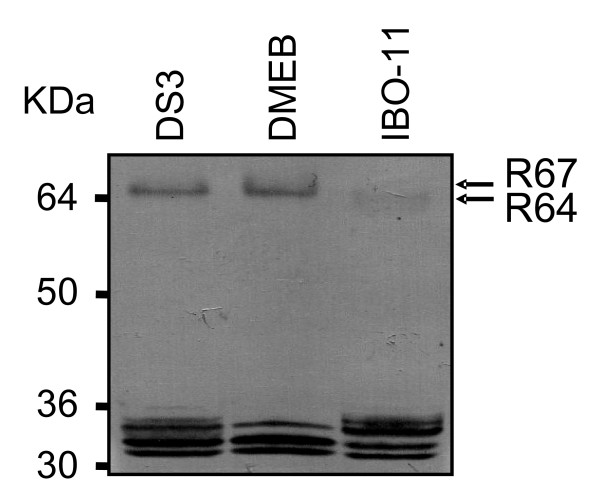
**Immunoblotting with the specific anti-R67 or anti-R64 antibody**. Protein extracts of *Ae. aegypti *MG from the *DS3*, *DMEB *and *IBO-11 *strains were separated by SDS-PAGE, transferred to PVDF membranes and immunoblotted with the specific anti-R64 antibody from *IBO-11*. Actin was used as control of the amount of protein loaded on each lane. Similar results were observed when anti-R67 from *IBO-11 *or anti-R67 or anti-R64 from *DS3 *was used.

To determine whether the 67 and 64 kDa MG proteins from the three *Ae. aegypti *strains are related, we obtained specific polyclonal antibodies against those proteins from the *DS3 *and *IBO-11 *strains and used them for immunoblotting of SDS-PAGE. Both the 67 and 64 kDa proteins were recognized by the antibodies. Figure 3 shows an immunoblot with the specific antibody against R64 from the *IBO-11 *strain; equivalent results were observed when we used the other antibodies. The main protein recognized by the antibodies in *DS3 *and *DMEB *had an apparent molecular weight of 67 kDa. The *IBO-11 *strain displayed a protein with apparent molecular weight of 64 kDa, although the band was very faint, probably because its relative concentration was lower than in the other two strains.

Thus, we found that the differences observed by VOPBA (Figure 1A) were not due to differences in the affinity of R67 and R64 for DENV, but may have been due to differences in the amounts of R67 and R64 in MG from each strain. Each specific band in the membranes was analyzed by densitometry and normalized to actin as control of the quantity of protein loaded on to each lane.

### Distribution of R67/R64 in *Aedes aegypti *midgut

**Figure 4:**
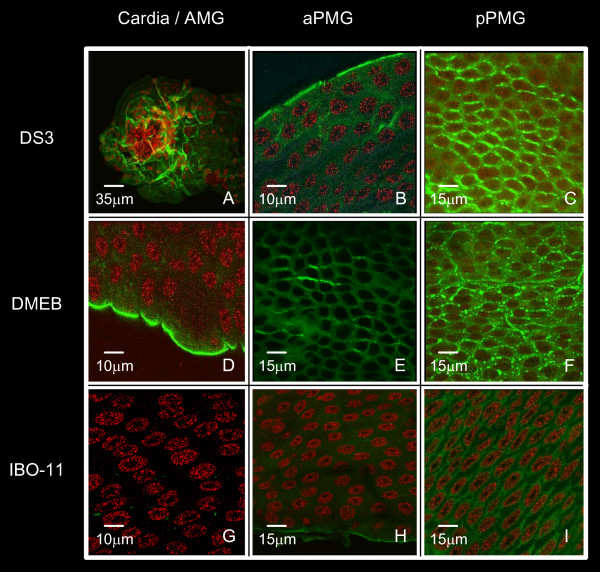
**Immunofluorescence with the specific anti-receptor antibody**. MG dissected from the *DS3*, *DMEB *and *IBO-11 *strains were immunolabeled with the specific anti-R67 antibody from *IBO-11 *and analyzed by confocal microscopy. Strains: *DS3 *(panels A, B, C), *DMEB *(panels D, E, F), *IBO-11 *(panels G, H, I); analyzed region: cardia/AMG (panels A, D, G), aPMG (panels B, E, H), pPMG (panels C, F, I). AMG (anterior midgut), aPMG (anterior segment of the posterior midgut), pPMG (posterior segment of the posterior midgut).

To determine the distribution of R67 and R64 along the MG in the *DS3*, *DMEB *and *IBO-11 *strains, we performed confocal microscopy on dissected MG, immunofluorescently labeled with the specific antibody against those proteins (Figure 4). This revealed the distribution of the proteins between the cells facing the lumen and the basal lamina (BL) of the epithelium, and from the cardia through the anterior MG (AMG) and posterior MG (PMG). The proteins were detected in the BL all along the MG in all three *Ae. aegypti *strains (evident in panels 4B, 4D and 4H). In addition, the specific antibody bound to epithelial cells beside the cell membrane in all strains from the middle to the end of the PMG (pPMG); in this region the fluorescence was more intense in *DS3 *and *DMEB *MGs (4C, 4F) than in *IBO-11 *MG (4I). These results suggest a higher density of R67 and R64 in *DS3 *and *DMEB *MGs. Control MGs stained with pre-immune serum showed no-fluorescence (results not-shown).

### Time-course of DENV infection in *Aedes aegypti *midgut

We analyzed the time-course of infection all along the MG in order to determine the density and distribution of DENV and its binding proteins at the outset of the infective process.

**Figure 5:**
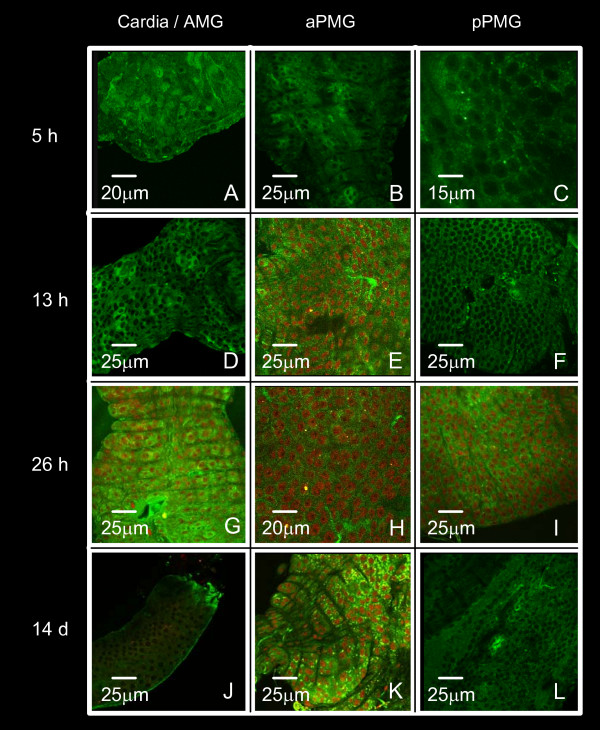
**Time-course of midgut DENV infection in *Ae. aegypti DS3 *strain**. Immunofluorescent labelling with the specific antibody anti-DEN-2 protein E was followed by confocal microscopy. MGs were dissected from *DS3 *mosquitoes 5, 13, 26 h and 14 d after infection. Cardia/AMG (panels A, D, G, J), aPMG (panels B, E, H, K), pPMG (panels C, F, I, L).

**Figure 6:**
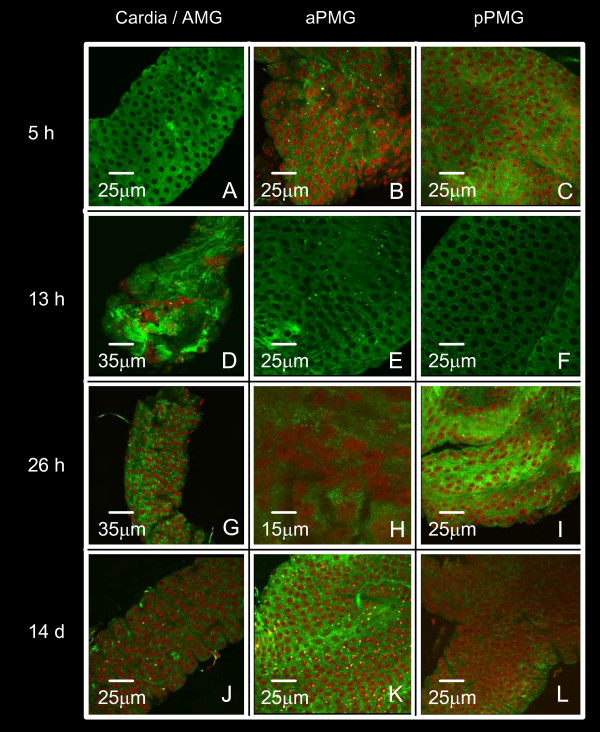
**Time-course of DENV infection in *Ae. aegypti *midgut (*DMEB *strain)**. Immunofluorescent labeling with the specific antibody anti-DEN-2 protein E was followed by confocal microscopy. MGs were dissected from *DMEB *mosquitoes 5, 13, 26 h and 14 d after infection. Cardia/AMG (panels A, D, G, J), aPMG (panels B, E, H, K), pPMG (panels C, F, I L).

**Figure 7:**
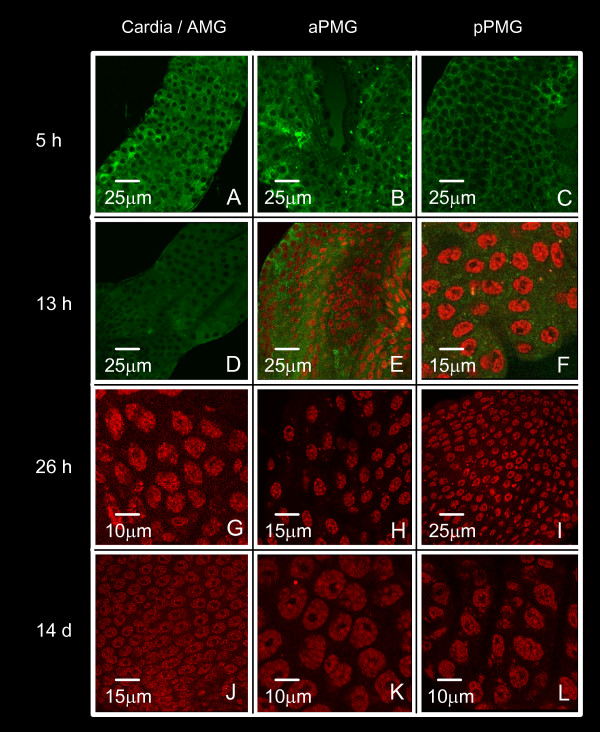
**Time-course of DENV infection in *Ae. aegypti *midgut (*IBO-11 *strain)**. Immunofluorescent labeling with the specific antibody anti-DEN-2 protein E was followed by confocal microscopy. MGs were dissected from *IBO-11 *mosquitoes 5, 13, 26 h and 14 d after infection. Cardia/AMG (panels A, D, G, J), aPMG (panels B, E, H, K), pPMG (panels C, F, I L).

Mosquitoes were infected via an artificial membrane feeder and the entire MG was dissected at 5, 13, 26 h and 14 days of cultivation after infection. The MG was examined from the cardia through the AMG and PMG by confocal microscopy after immunofluorescent labeling with anti-DEN-2 protein E. Figures 5, 6 and 7 correspond to the *DS3*, *DMEB *and *IBO-11 *strains respectively.

DENV reached the epithelial cells in all strains 5 h after infection, before the peritrophic matrix (PM) was formed. The distribution of DENV in the *IBO-11 *strain was similar to the other strains until 5 h, but virus density decreased with incubation time; the fluorescence had very low intensity at 13 h after infection; and at 26 h and 14 days after infection it had completely disappeared in 80% of the analyzed MGs.

In the *DS3 *and *DMEB *strains the fluorescence was very similar in each region analyzed at all times after infection; the infection increased with time and viral envelope antigen was apparent in the BL from 5 h until 14 days after infection irrespective of the distribution in epithelial cells (evident in panel 5J).

**Figure 8:**
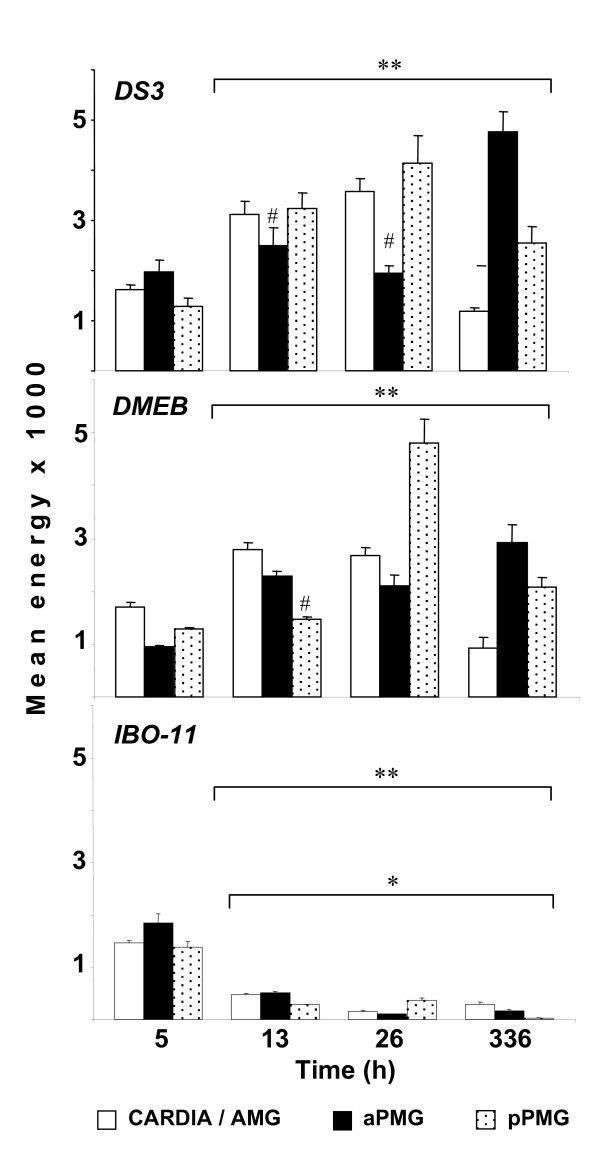
**Time-course of DENV infection in *Ae. aegypti *midgut**. Viral envelope antigen distribution during the DENV infection was analyzed in the MG of the *DS3 *strain (A); *DMEB *strain (B); or *IBO-11 *strain (C) in the Cardia/AMG (anterior midgut), aPMG (anterior segment of the posterior midgut), pPMG (posterior segment of the posterior midgut). Values are expressed as the mean ± S.E.M. ANOVA followed by All Pairwise Multiple Comparison Procedures (Student-Newman-Keuls Method) (p < 0.05); n = 4 independent replications included at least 5 mosquitoes for each strain and each infection time point. Values from *DS3 *or *DMEB *strains were compared with *IBO-11 *strain at 13, 26 and 336 h in each MG region (*p < 0.05, significantly different); Cardia/AMG, aPMG, or pPMG values from *DMEB*, *DS3 *or *IBO-11 *strains at 5 h post-infection were compared with those post-infected for 13, 26 and 336 h in the respectively strains (**p < 0.05, significantly different; ^#^p > 0.05, non-significantly different).

We quantified the fluorescence (Figure 8) as mentioned in methods and noticed that the initial level was very similar in all three strains. In *DS3 *and *DMEB *the maximum level was very alike, containing no statistically significant difference after 26 h of infection at pPMG; after 14 days, this region showed less viral envelope antigen and virus was observed at the anterior PMG. Virus infection was higher at this time compare to infection after 5 h (p < 0.05). If we compare virus infection of *IBO-11 *strain with *DS3 *or *DMEB *at 13, 26 and 336 h the difference is very evident (*p < 0.05); contrary to infection at 5 h. Furthermore, if we compare, infection at 5 h with all times in each strain, we observe that *DS3 *and *DMEB *showed an increase (**p < 0.05); opposite to the *IBO-11 *strain where there is an infection decrease (**p < 0.05).

Importantly, the highest fluorescence intensity observed in the MG at the beginning of infection (26 h after infection) was very similar to that observed by immunofluorescence assays using anti-R67/R64 antibody. All infected or non-infected MGs stained with pre-immune serum showed no-fluorescence (results not-shown).

## Discussion

The relationships between DENV and its arthropod vector *Ae. aegypti *are crucial, and analysis of host cell responses to flavivirus infection of mosquito vectors is particularly important for understanding the maintenance and transmission of the disease. Mosquito populations differ in their susceptibility to flavivirus development, termed "vector competence", reflecting the different barriers encountered by the virus from its entry into the mosquito to its release in the saliva. Factors such as specific mosquito receptors on the epithelial cells as well as differential viral replication in the mosquito are critical for vector competence in addition to other genes as has been exhibited by the QTL studies [39]. In the laboratory we have three mosquito strains with different susceptibilities to DENV infection (*DS3*, *DMEB *and *IBO-11*) and these have allowed us to determine whether MG cell receptors for DENV may be markers of vector competence.

A possible explanation for the wide spectrum of DENV receptors in host cells may be that different cell types have been used; alternatively, the reported proteins may not all be cell membrane components. Increasing evidence [33,40] suggests that DENV interacts differently with mammalian and mosquito cells; accordingly, we have studied the molecules that serve as true DENV receptors in the MG of *Ae. aegypti*. We previously showed by VOPBA [29] that the four serotypes of DENV mainly recognized two membrane proteins with apparent molecular weights of 80 (R80) and 67 (R67) kDa in *Ae. albopictus *C6/36 cells and *Ae. aegypti *MG.

Both of these receptors were detected in the MG of strains *DS3*, *DMEB *and *IBO-11 *in this study, although *IBO-11 *displayed a protein with a slightly lower apparent molecular weight (64 kDa) in addition to the 67 kDa protein, and the quantity of R67 was much lower than in the other two strains. R67 and R64 seem to be the same protein, because both are recognized by polyclonal anti-R67 and anti-R64 antibodies from the *DS3 *and *IBO-11 *strains. The size difference was also detected by two dimensional gels (Figure 2). Munoz et al. [24] and Mercado-Curiel et al. [29] showed that anti-R67 antibody inhibited DENV infection more strongly than anti-R80 in C6/36 cells. This is in agreement with the present results, since R80 was recognized in all three strains with no apparent change, suggesting that although this protein participates in DENV binding; its role may be different. It will be necessary future studies to determine R80 function during mosquito virus infection. Difference between R67 and R64 will be the objective of future studies, since they may be attributable to many facts such as post-translational modifications, different splicing or a deletion in the coding region.

Studies of the vector competence of *Ae. aegypti *for DENV are typically qualitative, involving the detection of viral antigens in the MG and the head. This is the first study to scan DENV infection and R67 protein distribution thoroughly along the entire MG, taking into account that MG contains morphologically different epithelial cells [41] with different biochemical characteristics [42,43]. Previous studies have not determined DENV distribution in detail; most of them have concerned virus titer (presence or absence) in the whole mosquito MG [44-49].

R67/R64 were not homogeneously distributed over the epithelial cells along the MG in the three strains; they were mainly present in the cell membrane and behind the intercellular spaces in the region named pPMG, which extends from middle to the end of the posterior MG in the neighborhood of the hindgut. The quantity of R67/R64 in the pPMG region was noticeably higher in *DS3 *and *DMEB *than in the *IBO-11 *strain (Figure 4). The receptor distribution was similar to the distribution of infection at the outset. Moreover, we observed a correlation among receptor abundance, presence of DENV and mosquito strain susceptibility (Figures 4 and 8).

Analysis of the time course of DENV infection from the beginning to 14 days gave insight into the earliest events in DENV infection of the MG in the three strains. MG infection diminished by 14 days post-infection, as described previously [47]. We also observed that MGs from the different strains showed a difference in the degree of infection, depending on the susceptibility of the strain. For example, the *IBO-11 *strain showed almost no fluorescence after 26 days.

Interestingly, DMEB strain showed increase in infection up to the 26 h in all the three MG regions infection, having the maximal virus accumulation in the pPMG and then diminished by 336 h post-infection compare to the susceptible strain *DS3 *that have the maximal virus accumulation in aPMG at 14 days post-infection. These results also display a statistically significative MG infection increase from the first 5 h post-infection to 26 h in *DS3 *and *DMEB *strains (**p < 0.05). Moreover, *IBO-11 *strain exhibited a significative decrease (**p < 0.05) of MG infection from 5 to 336 h post-infection. Furthermore, virus infection of *IBO-11 *strain was almost completely abolish (*p < 0.05, Figure 8) compared to *DS3 *and *DMEB *strains from 13 to 336 h post-infection.

Recently, the *Ae. aegypti *genome has been sequenced [50]. This will facilitate the identification of genes encoding the R67/R64 DENV receptors, which could be important for influencing the MG infection barrier (MIB). This is in agreement with Miller and Mitchell [51], who showed that susceptibility, depends on multiple genes. They selected refractory or highly susceptible strains and obtained progeny with intermediate susceptibility, which suggests that alleles at vector competence loci act additively. Bosio et al. [14] proposed a significant additive genetic effect in MIB and demonstrated that the DENV titer in the MG and head did not correlate with the rate of infection. They also showed that the heritability for virus titer in tissues (MG or head) were almost identical in different strains of *Ae. aegypti *formosus; and showed that the amount of virus in the MG did not determine if virus was disseminated, which hypothetical may be due to the presence or absence of DENV receptors. In the present study we also observed similar maximum levels of DENV in the *DS3 *and *DMEB *strains, suggesting that genes that influence the virus titer have minimal impact on overall vector competence.

Further studies are needed to explain the fact that the *DMEB *strain, which has MEB, showed the presence of DENV in the BL all along the MG throughout the time course examined. The lack of infection in peripheral tissues may have been mainly due to the lack of cell receptors, or to additional factors specific to the kind of cell that allows DENV to infect those organs.

*Ae. aegypti *seems to be the most vulnerable link in the transmission chain; at present there is no genetic marker of vector competence for DENV. Such a marker would be very important in the design of new mosquito control strategies, such as campaigns focused on natural populations that could be easily identified by their high DENV susceptibility, in order to prevent dengue epidemics.

## Conclusion

Our results may suggest that R67/R64 from the *Ae. aegypti *MG could be used as a vector competence marker, since those proteins are the main ones involved in the recognition of DENV by MG cells. The amount of these proteins in the MG varied proportionally to mosquito vector competence and their distribution along the MG correlated with the distribution of DENV infection.

## Methods

### Virus

DEN-2 Jamaica strain was expanded in Vero cells, purified from the culture supernatants as previously described [52,53] and kept frozen at -70°C pending for use. Briefly, Vero cells were cultured at 37°C, 2% CO_2 _in Dulbecco's Modified Eagle's Medium (DMEM; HyClone, Logan, Utah, USA) supplemented with 5% heat-inactivated fetal bovine serum (FBS; Gibco BRL, Gaithersburg, MD, USA), 100 units/ml of penicillin and 100 μg/ml of streptomycin. Vero cells were infected with 0.2 ml of DEN-2 inoculum with an input MOI of 600 PFU/plate and incubated for 10 days; DEN-2 was purified from the clarified supernatants by a 30/60% sucrose step gradient. Titers of virus stocks made in LLC-MK2 cells [52] were 8 × 10^8 ^PFU/ml.

### Mosquito culture

Mosquitoes (*DS3*, *DMEB *and *IBO-11 *strains) were laboratory-reared and maintained at 28°C and 80% RH with a 12 h photoperiod using standard mosquito-rearing procedures [54]. They were fed with blood meals to maintain the strains and the eggs were collected in water cups containing paper filters.

### Mosquito infection and midgut dissection

Mosquitoes (*DS3*, *DMEB *and *IBO-11 *strains) were infected via an artificial membrane feeder [55]. Briefly, the blood meal consisted of equal parts of virus suspension, washed sheep erythrocytes and FBS in 10% sucrose. It was incubated at 38°C for 15 min, then placed in membrane feeders covered with hog gut and maintained at a constant temperature of 37°C. Mosquitoes (250, 3–4 days old) were starved of sucrose and deprived of water for 30 hours prior to blood feeding. They were allowed to feed for 45–60 minutes. Fully engorged females were selected and held in the insectary.

The entire MG was dissected from 25 mosquitoes at 5, 13, 26 h and 14 days after feeding. The procedure was carried out in 10 μl phosphate buffered saline (PBS). The MG was rinsed twice and resuspended in 10 μl of PBS. MGs were immunolabeled with a specific antibody against DEN-2 envelope protein E (anti-DEN-2 protein E).

All experiments included control mosquitoes fed with a blood meal without virus. MGs from non-infected mosquitoes were also used to obtain protein extracts and were examined by immunofluorescence with anti-R67.

### DEN-2 affinity chromatography

Purified DEN-2 was bound covalently to 1 g CNBr-activated Sepharose™ 4B (Amersham Pharmacia Biotech) as described previously [29]. The DEN-2-Sepharose™ 4B column was either used immediately or stored in 0.002% sodium azide at 4°C pending for use.

Protein extracts were obtained by homogenizing MGs (300/ml from the *DS3 *and *IBO-11 *strains) in buffer E (50 mM Tris-HCl, pH 7.2, 1 mM EDTA, 0.05% v/v Triton X-100) containing a protease inhibitor cocktail (Sigma-Aldrich P8340). To obtain the soluble proteins, the homogenate was centrifuged for 10 min at 10,000 rpm at 4°C.

Each MG protein extract (1 mg) was applied to the DEN-2-Sepharose™ 4B column (1 ml) equilibrated in buffer E and washed with the same buffer. The DEN-2 binding proteins were eluted with buffer E containing 0.5 M NaCl. Fractions of 0.5 ml were collected and the protein content was monitored by the Bradford method [56]. The retained and eluted proteins were precipitated with acetone, resuspended in a 10 μl buffer E/protease inhibitor cocktail (Sigma-Aldrich P8340) and analyzed by 10% sodium dodecylate sulfate polyacrylamide gel electrophoresis (SDS-PAGE) [57].

### Preparation of specific polyclonal antibodies against DEN-2 protein E, R67 and R64

To obtain specific anti-DEN-2 protein E, 10% SDS-PAGE was performed with purified DEN-2 and the corresponding 52 kDa band from the silver-stained gel was excised and used to immunize BALB/c mice as described below.

Proteins retained and eluted from the DEN-2-Sepharose™ 4B column were separated by 10% SDS-PAGE. After silver staining, the 64 or 67 kDa band was excised, cut into small pieces, suspended in PBS and mixed with an equal volume of Titer-Max adjuvant (CytRx Vaxcel Inc., Norcross, GA) to immunize two groups of BALB/c mice and obtain specific antibodies against R67 or R64 from *DS3 *or *IBO-11 *strain *Ae. aegypti *MGs.

Pre-immune sera were obtained before immunization. The mice received a booster fifteen days after the first immunization. They were bled after thirty days and the sera were stored at -70°C until use. Negative controls using pre-immune sera were included in all assays. All immunofluorescence and immunoblotting results were reproducible using either anti-R67 or R64 from either *DS3 *or *IBO-11*.

### Immunofluorescence with anti-DEN-2 protein E and anti-R67

After the MGs were dissected from the different strains and different infection times, including non-infected mosquitoes as negative control; they were fixed for 2 h in 4% p-formaldehyde (Sigma-Aldrich Corporation), washed with PBS, 0.2% PBT (5% BSA, 0.2% Triton X-100 in PBS), incubated overnight at 4°C with anti-DEN-2 protein E diluted 1:75 or anti-R67 diluted 1:40, washed with 0.1% PBT (0.1% Triton X-100), incubated for 2 h with 1:500 FITC goat anti/mouse (Zymed Laboratories Inc. S. San Francisco, CA USA), stained with 1 μg/ml propidium iodide (Sigma-Aldrich Corporation), and then washed with 0.1% PBT. Finally, each MG was placed individually on a glass slide with a Vecta Shield (Vector Laboratories). All preparations were examined by confocal microscopy (TCS SP5 Leica Microsystems).

### Confocal microscopy analysis

Confocal Image was captured using a Leica confocal microscope scanning system. The confocal microscope was set up for all the experiments as follow: speed 400 Hz, PMT1-746, PMT2-933, potency 1/3 and laser 26%. Fluorescence evaluation was made using a 64× (NA 0.3) objective. The lens is raised to its maximum specified height. The detector is secured on the stage and centered grossly using either laser fluorescent or light. The detector position is then adjusted more accurately to achieve maximum signal intensity by using the microscope's x/y joystick. The CLSM zoom factor is set from 8 to 32 to reduce the beam scan and to focus it into the "sweet spot" of the detector.

A series of 30 successive sections were recorded along the optical axis of the microscope over a range of the specimen planes with a depth separation of 1 μm for each one of the three different MG regions (Cardia/AMG, aPMG, pPMG) and mosquito strains, comprising a total of 3 series of 30 slices. Then each confocal fluorescence images was divided in areas of 1000 μm2. This was done for every time and each mosquito strain.

The Leica software (1997–2002, Leica Microsystems Heidelberg GmbH) was used to evaluate all images. ANOVA statistic analysis was conducted for all the data.

Results represent the analysis of at least 20 MGs for each infection time and for each mosquito strain in four independent experiments.

### Statistical analysis

The means and standard errors of means (S.E.M.) were calculated for all groups. The data was subjected to a two-way analysis of variance (ANOVA) by General Linear Model followed by All Pairwise Multiple Comparison Procedures (Student-Newman-Keuls Method) to determine whether means were significantly different among them. All P values less than 0.05 were considered to indicate statistical significance. All the statistics were carried out in SigmaStat 2.03 software and data plotted in SigmaPlot 2001.

### 2D gel electrophoresis

Midguts (300) from each strain were solubilized in 125 μl of IPG strip rehydration buffer (8 M urea, 2% CHAPS, 10 mM DTT, 0.2% Bio-Lyte) at room temperature and centrifuged at 10,000 rpm for 10 min. The resulting supernatants, containing approximately 936 μg protein for *DS3*, 1080 μg for *DMEB *and 1143 μg for *IBO-11*, were used to rehydrate each ReadyStrip IPG, pH range 3–10, 7 cm (BioRad Laboratories, Hercules, CA, USA) under passive conditions overnight at 4°C in the focusing tray (Mini-PROTEAN 3 cell). Subsequently, IEF was carried out as recommended by the manufacturer (2 h at 4000 V with a gradient until a total of 10000 volt-hour was reached).

The strips were removed from the focusing tray and incubated for 15 min in 1 ml equilibration buffer I (50 mM Tris-HCl, pH 8.8, 6 M urea, 2% SDS, 30% glycerol, 1% DTT) and 15 min in equilibration buffer II (6 M urea, 0.375 M Tris-HCl, pH 8.8, 2% SDS, 20% glycerol, 2.5% w/v iodoacetamide). They were washed with distilled water and placed on the top of the second dimension gel (10% SDS-PAGE). Molecular weight markers were applied to small pieces of chromatography paper and inserted next to each strip on the top of the gel, then the strips and markers were sealed with ReadyPrep Overlay Agarose (0.5% low melting point agarose in 1X Tris/Glycine/SDS and 0.003% Bromophenol Blue). The second dimension was developed at a constant 150 V (Mini Protean 3, BioRad Laboratories, Hercules, CA, USA). The gels were silver stained or used for 2D VOPBA as described below.

### 1D and 2D virus overlay protein binding assay (VOPBA)

MG protein extracts (35 μg) from each strain, or 72 μg for *DS3*, 59 μg for *DMEB *and 155 μg for *IBO-11*, were obtained as mentioned above by DEN-2 affinity chromatography and separated by 10% SDS-PAGE. Two dimensional gels were developed with approximately 1000 μg protein from MGs of each strain. All the gels were blotted on to polyvinylidine difluoride (PVDF) membranes using the WetBlot technique (BioRad Laboratories, Hercules, CA, USA) in transfer buffer (15.6 mM Tris-HCl, 120 mM glycine, 20% methanol). After transfer was complete, VOPBA was performed as previously described [24]. Briefly, the membrane was blocked for 1 h at room temperature with 5% skim milk in TBS (100 mM Tris-HCl, 0.15% NaCl), incubated overnight with purified DEN-2, washed with TTBS (100 mM Tris-HCl, 0.15% NaCl, 0.2% Tween 20) and incubated overnight with 1:200 anti-DEN-2 protein E at 4°C. Mouse anti-rat brain actin Mab (generously provided by Dr. Manuel Hernandez-Hernandez from the department of Cell Biology, Centro de Investigación y de Estudios Avanzados del IPN, Mexico) diluted 1:50 was also included in the VOPBA from 1D gels. The epitope recognized by this MAb is conserved among all actins. After washing with TTBS, the membrane was incubated for 2 h at room temperature with 1:1000 alkaline phosphatase (AP) labeled goat anti-mouse (Zymed Laboratories Inc. S. San Francisco, CA USA) and the reactive proteins were visualized by color development with the chromogenic substrate BCIP/NBT.

### Immunoblotting with anti-R67 and anti-R64

MG protein extracts from the *DS3*, *DMEB *and *IBO-11 *strains were separated by 10% SDS-PAGE, transferred to PVDF membranes (BioRad Laboratories, Hercules, CA, USA) and incubated with anti-R67 or anti-R64 diluted 1:75 in TBS (100 mM Tris-HCl, 0.15% NaCl). In addition, anti-actin antibodies diluted 1:50 in TBS were used. The membranes were incubated for 2 h at room temperature with the secondary antibody, AP goat anti-mouse (Zymed Laboratories Inc., San Francisco, CA, USA) diluted 1:1000, and color was developed as recommended by the manufacturer and described above.

## Authors' contributions

RFMC carried out all the experiment assays. MLM, RFMC and WCB participated in the discussion of results. RFMC and MLM contributed to assembling the manuscript. MLM proofread the manuscript. All authors participated in the discussion of results, read and approved the final manuscript.
